# Aortic stiffness in patients with hypertension and ascending aortic aneurysm

**DOI:** 10.1590/1806-9282.20260687

**Published:** 2026-08-10

**Authors:** Senol Yavuz, Mesut Engin

**Affiliations:** 1 University of Health Sciences, Bursa Yuksek Ihtisas Training and Research Hospital, Department of Cardiovascular Surgery – Bursa, Türkiye.

Dear Editor,

We read with great interest the article by Arpa et al.^1^, demonstrating a potential relationship between the development of ascending aortic aneurysm (AscAA) and increased aortic stiffness in hypertensive patients. The authors have made a significant contribution to our cardiovascular practice with this study. We would like to congratulate the authors for bringing this topic to our attention. However, we believe some important ­methodological and conceptual points require further discussion.

AscAA carries a risk of complications that can lead to fatal outcomes, such as rupture and/or dissection. Although an increased aortic diameter suggests an indication for elective aortic repair, complications generally occur at diameters below the surgical threshold. Aortic stiffness and expandability are considered as alternative risk markers^2^.

The inconsistency in device terminology in the authors’ article is noteworthy. While the authors use the phrase “oscillometric arteriograph device” in the title of the article, the Methods section states that the measurements were taken with a Mobil-O-Graph PWA (I.E.M. GmbH, Germany) device. Arteriograph (TensioMed, Hungary) and Mobil-O-Graph devices are two independent oscillometric platforms that use different algorithms and can produce differing measurement results. Benas et al. reported an average difference of up to 9.2 mmHg between these two devices, particularly in central systolic blood pressure measurements^3^. This makes data reproduction difficult and significantly reduces interpretability.

Numerous studies have shown that pulse wave velocity (PWV) is an independent predictor of cardiovascular events and mortality. The coefficient of variation for PWV between measurements with Arteriograph and Mobil-O-Graph devices has been reported to be 9.5%^3^. The authors, in their current study, noted that while augmentation index (Aix) was significantly higher in the AscAA group, they found no difference between the groups in terms of PWV^1^. It would be expected that they would discuss in detail whether this result is related to device sensitivity, a technical limitation of the measurement method, sample size, age distribution, blood pressure level, or antihypertensive treatments. On the other hand, Lindenberger et al. have shown that regional and global PWV assessment in aortic diseases reveals significant differences in larger samples^4^. To assess whether the difference observed in PWV (8.85 vs. 8.59 m/s) in the authors’ current study would be considered significant by clinicians, it would be appropriate to define the minimal clinically important difference.

The authors measured the ascending aortic diameter using 2D transthoracic echocardiography in the study and noted its limitations compared to coumpted tomography (CT). In thoracic aortic pathologies, measurement differences between imaging modalities play a critical role in aneurysm classification and growth rate assessment. Echocardiography-based measurement can be limiting in terms of both accuracy and reproducibility, particularly at the 40 mm threshold in echocardiography, where patient classification can be negatively affected. Echocardiographic definition of aneurysm varies according to gender. It is defined as an aortic diameter higher than the gender-specific threshold values at the level of Valsalva sinus (≥39 mm for women, ≥44 mm for men) or at the level of the ascending aorta (≥42 mm for women, ≥46 mm for men)^5^. We did not see this distinction in the study.

In conclusion, the authors’ study makes a significant contribution by demonstrating increased aortic stiffness in the presence of AscAA in hypertensive patients; however, the ­findings should be interpreted cautiously due to the measurement method, sample size, and cross-sectional design. It is important that future studies should include elements such as CT-based confirmation, multi-device validation, and longitudinal follow-up.
